# Ipsilateral radial nerve, median nerve, and ulnar nerve injury caused by crush syndrome due to alcohol intoxication: A case report

**DOI:** 10.1097/MD.0000000000017227

**Published:** 2019-09-20

**Authors:** Yuan-Wei Zhang, Cheng Ju, Xue-Lei Ke, Xin Xiao, Yan Xiao, Xi Chen, Su-Li Zhang, Hong-Yan Ge, Liang Deng

**Affiliations:** a Department of Orthopedics, Jiangxi Provincial People's Hospital Affiliated to Nanchang University; b Medical Department of Graduate School, Nanchang University, Nanchang; c Department of Operating Room, Wujin Hospital Affiliated to Jiangsu University, Changzhou, Jiangsu, China.

**Keywords:** autogenous compression, median nerve, peripheral nerve injury, radial nerve, ulnar nerve

## Abstract

**Rationale:**

Autologous peripheral nerve injury caused by crush syndrome due to alcohol intoxication is relatively rare, and to our knowledge, the compression of 3 upper limb nerves at the same time has not been reported previously. If a compressive peripheral nerve injury is not treated in a timely manner, it is difficult to recover neurological function, and the prognosis is poor.

**Patient concerns:**

Here, we present a case of a 50-year-old man with ipsilateral radial nerve, median nerve, and ulnar nerve injuries caused by autogenous compression after drunkenness.

**Diagnosis:**

Electromyography and nerve conduction studies suggested peripheral nerve injury in the left upper limb. The diagnosis was injury to the radial nerve, median nerve, and ulnar nerve in the left upper arm.

**Interventions:**

Exploratory neurolysis surgery of the radial nerve, median nerve, and ulnar nerve was performed in the left upper arm. Postoperative oral neurotrophic drugs were administered, and functional exercise was performed.

**Outcomes:**

After timely diagnosis and treatment, the strength of the left upper arm muscle recovered, and the prognosis of neurological function was satisfactory during 3 years of follow-up sessions.

**Lessons:**

In the treatment of such patients, a comprehensive understanding of their medical history and a strict physical examination should be performed. Combined with neuroelectrophysiological and imaging examination, the diagnosis can be confirmed. After timely diagnosis and treatment, the prognosis is mostly excellent.

## 1 Introduction

Autologous compression of a peripheral nerve injury occurs mostly in drunkenness, coma, or sleep. The affected limb is compressed under the body, which leads to limb numbness and limited function. According to previous reports, due to the characteristics of anatomy and pathogenic factors of the radial nerve, it is the most common compressive nerve injury of the upper limbs.^[1]^ However, the compressive injury of median nerve and ulnar nerve has rarely been reported, and the cases of simultaneous injury to all 3 nerves are especially uncommon. Here, we describe a rare case of ipsilateral radial nerve, median nerve, and ulnar nerve injury caused by crush syndrome due to alcohol intoxication.

## 2 Case presentation

A 50-year-old Chinese man was initially admitted to our department for the sensorimotor dysfunction in left forearm, wrist, and hand. Before that, the patient had visited a clinic and been treated with oral neurotrophic drugs. Conservative treatment was ineffective, and the symptoms gradually worsened. Tracing his past history, we found that he was accustomed to resting in the left lateral position, with the left upper limb pressed under his body. After an episode of drunkenness 2 months ago, he rested in the left lateral position, with the left upper limb pressed under his head for more than 6 hours. After waking, he showed dysfunction when extending the left wrist and fingers, weakness of the left upper limb and inability to grasp.

**Figure 1:**
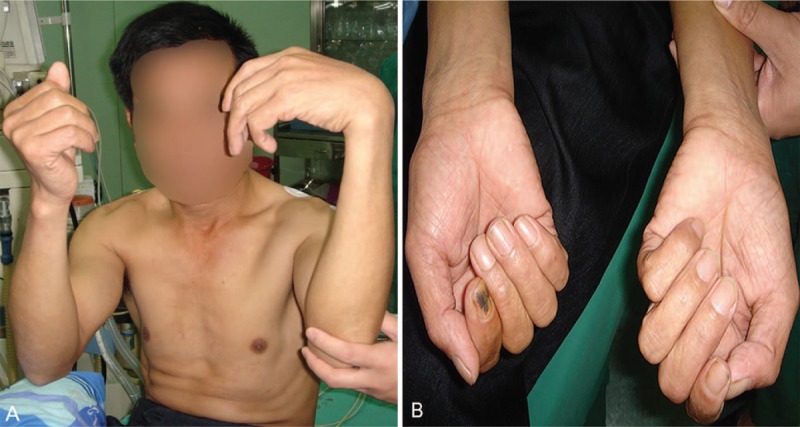
The symptoms of a 50-year-old man who suffered from injury of the radial nerve, median nerve, and ulnar nerve of his left upper limb due to the autogenous compression after drunkenness. (A) The fingers and wrist of the left hand were dropped, and presented as a “claw-shaped hand” deformity. (B) The left forearm flexor muscles group, major and minor thenar were apparently atrophied compared with the contralateral side.

Physical examination revealed that the fingers and wrist of his left hand were dropped and presented as a “claw-shaped hand” deformity. The left forearm flexor muscles group, the major and minor thenar, was characterized as muscle atrophy (Fig. 1). The sensory function of the dorsal side of the left forearm, the major and minor thenar area and the tiger's mouth area was decreased. Moreover, the motorial function of the left thumb against the palm was absent. In addition, the muscle strength of the left upper limb was apparently decreased, the left brachioradialis muscle strength was grade 0, the ulnar wrist flexor muscle strength was grade 3, the wrist and finger flexor muscle strength were grade 4, and the wrist and finger extensor muscle strength was grade 0. The clipping paper test of the left thumb and index finger was positive, while the Hoffmann test was negative.

Electromyography (EMG) and nerve conduction study (NCS) were rapidly performed after admission, indicating peripheral nerve injury of the left upper limb. EMG showed the fibrillation potential in the left short abductor muscle of the thumb at rest. The amplitude and latent period of the action potential were normal in light contraction, and presented as mixed phase in heavy contraction. NCS revealed the decreased amplitude of sensory nerve action potential in both the left radial nerve, median nerve and ulnar nerve, and sensory nerve conduction velocity slowed down apparently. Meanwhile, the cranium and cervical vertebra magnetic resonance imaging (MRI) was performed to exclude intracranial and cervical lesions, and the results were normal. Consequently, the patient was diagnosed with radial nerve, median nerve, and ulnar nerve injuries in the left upper arm caused by autogenous compression after drunkenness.

**Figure 2:**
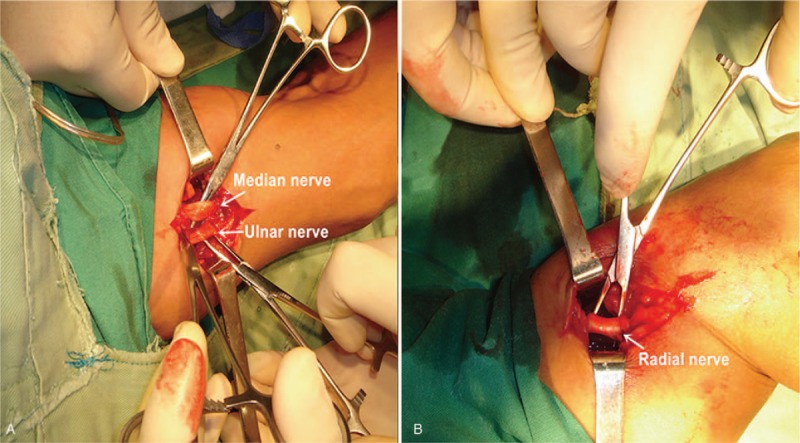
Images from the exploratory neurolysis surgery of the radial nerve, median nerve, and ulnar nerve in the left upper limb. (A) The median nerve and ulnar nerve (indicated by the arrow) were observed to be whitened, and have edema and adhesion. (B) The radial nerve (indicated by the arrow) was whitened, and had edema and adhesion due to prolonged compression.

After definite diagnosis, the patient underwent exploratory neurolysis surgery of the radial nerve, median nerve, and ulnar nerve in the left upper limb. Under brachial plexus block anesthesia, the exploration incision of the radial nerve, median nerve, and ulnar nerve in the left upper arm was selected respectively according to the injured site. In the operation, 3 nerves were observed to be whitened, and have edema and adhesion (Fig. 2). The adhesions were released separately. Postoperative oral neurotrophic drugs were continued and the patient was guided to perform passive thumb and finger extension exercises. During 3 years of follow-up, the left upper limb sensorimotor function recovered well. The left brachioradialis muscle strength was grade 5, the ulnar wrist flexor muscle strength was grade 5, the wrist and finger flexor muscle strength were grade 4, and the wrist and finger extensor muscle strength was grade 4. Moreover, the motorial function of the left thumb against the palm was restored, and the clipping paper test of the left thumb and index finger was negative.

## 3 Discussion

The microvasculature in the nerve is the terminal artery, and the peripheral nerve is adjacent to the skeleton in the process of accompanying the limbs. Therefore, once subjected to heavy compression, it is prone to cause compressive and ischemic injury, bringing about subperineurial and endoneurial edema and ultimately leading to the loss of nerve conduction function.^[2]^ After a long period of compression, hyperemia and edema of the peripheral nerve tissue may further aggravate the compression site, which results in local connective tissue hyperplasia and segmental demyelinating degeneration of myelinated fibers.^[3]^

Due to the characteristics of the anatomy and pathogenic factors of the radial nerve, that is, it runs along the radial nerve groove close to the dorsal side of the middle humerus, once a person falls asleep in an inappropriate position while intoxicated or sleep, the weight of the body may completely compress on the upper limb. This results in a decrease in blood supply in this area, followed by sensorimotor dysfunction. However, the median nerve and ulnar nerve are located deeper in the upper limb, so injuries to these nerves are more common in penetrating injuries from severe soft tissue injuries,^[4]^ and few cases have been reported in compressive nerve injury. In this case, we consider that the patient's habit of resting in the left lateral position, with the left upper limb pressed under his body, caused the latency of the compressive injuries. Moreover, unconscious left upper limb compression after drunkenness 2 months ago was the direct cause of the aggravation of the injury. Pressure on the contact area was large and maintained for a long time, and 3 nerves were simultaneously compressed, which resulted in the sensorimotor dysfunction after waking.

In terms of diagnosis, EMG combined with NCS contributed to clarifying the diagnosis, and the different electrophysiological changes exhibited helped to identify the location, nature, and degree of autogenous compressive peripheral nerve injury.^[5]^ In addition, the MRI combined with ultrasonography is helpful to determine the location and extent of nerve compression, and show the morphological changes of the nerves, providing an intuitive imaging basis for the nerve injury.^[6,7]^ However, since the causes of autogenous compressive peripheral nerve injury are not as obvious as traumatic injury, the neurological symptoms of the upper limb are easily confused with acute cerebral infarction and cervical spondylotic myelopathy, resulting in a certain rate of misdiagnosis.^[8,9]^ Therefore, in the treatment of such patients, it is essential to fully understand the medical history and carefully examine the body. When necessary, MRI of the cranium and cervical vertebra should be improved for the differential diagnosis.

In treatment, the first and most important step is to remove the compressive factors, and keep the affected limbs still and rested. Moreover, according to previous researches by He et al,^[10]^ the factors regarding the prognosis of nerve injuries included the age, gender, repair time, repair materials, and the defect length of nerve. Therein, the repair time played a vital role in determining the prognosis of nerve injuries, and the positive treatment within 3 months after the onset of neurological symptoms was recommended. Conservative treatment includes oral neurotrophic drugs, functional exercise of the affected limbs, and electrical stimulation therapy. The neurological function of some patients can be restored to normal after a period of conservative treatment. In general, the conservative treatment period for peripheral nerve injury is about 3 months. If there is no apparent improvement in symptoms beyond this time limit, surgery is considered.^[11]^ Generally, the therapeutic effect of surgery on nerve injury repair is that neurolysis is the best, followed by nerve anastomosis, while nerve transplantation is relatively poor.^[12]^ In this case, all 3 nerves were not severed, so only neurolysis was performed. Oral neurotrophic therapy and functional exercise were continued after the operation. During the 3 years of follow-up sessions, the neurological function of the patient recovered well.

## 4 Conclusion

Radial nerve injury is common in upper limb compressive nerve injury. However, we have described a rare case of ipsilateral radial nerve, median nerve, and ulnar nerve injury caused by crush syndrome due to alcohol intoxication. The diagnosis of compressive peripheral nerve injury mainly depends on strict physical examination, neurological examination, and corresponding imaging examination. If nerve function is not treated in good time, it is difficult to recover and the prognosis is poor. After timely diagnosis and treatment, the neurological function of this patient recovered well during 3 years of follow-up sessions.

## Author contributions

**Conceptualization:** Yuan-Wei Zhang, Cheng Ju, Xue-Lei Ke, Xin Xiao, Yan Xiao, Xi Chen, Liang Deng.

**Data curation:** Yuan-Wei Zhang, Cheng Ju, Xue-Lei Ke, Xin Xiao, Yan Xiao, Xi Chen, Liang Deng.

**Investigation:** Yuan-Wei Zhang, Cheng Ju, Xue-Lei Ke, Yan Xiao.

**Resources:** Yuan-Wei Zhang, Xue-Lei Ke, Xi Chen, Liang Deng.

**Supervision:** Yuan-Wei Zhang, Xin Xiao, Liang Deng.

**Validation:** Su-Li Zhang, Hong-Yan Ge, Liang Deng.

**Visualization:** Yuan-Wei Zhang, Su-Li Zhang, Hong-Yan Ge, Liang Deng.

**Writing – original draft:** Yuan-Wei Zhang, Cheng Ju, Liang Deng.

**Writing – review & editing:** Yuan-Wei Zhang, Cheng Ju, Su-Li Zhang, Hong-Yan Ge, Liang Deng.

Liang Deng orcid: 0000-0002-7221-9146.
